# Host and Microbe Scale Processes Jointly Shape Spatial Variation in Aphaenogaster (Hymenoptera: Formicidae)associated Wolbachia

**DOI:** 10.21203/rs.3.rs-8515672/v1

**Published:** 2026-01-29

**Authors:** Daniel Malagon, Benjamin Camper, Sophie Millard, Ernesto Recuero, Michael Caterino, Maslyn Greene, Anna Seekatz, Seth Bordenstein, Sarah Bordenstein, Sharon Bewick

**Affiliations:** Clemson University; Clemson University; Clemson University; Clemson University; Clemson University; Clemson University; Clemson University; Pennsylvania State University; Pennsylvania State University; Clemson University

**Keywords:** Aphaenogaster, Wolbachia, host-associated microbes, multiscale, environmental selection, dispersal limitation

## Abstract

The spatial distributions of host-associated (HA) microbes are shaped by the spatial processes of environmental selection and dispersal. However, unlike free-living organisms, HA microbes experience selection and dispersal at two separate spatial scales – the scale of the microbes and the scale of their hosts. Therefore, HA microbes must tolerate both the environment created by their host (microbe-scale environment) and the environment in which their host resides (host-scale environment). Likewise, HA microbes can disperse both between hosts through horizontal or vertical transmission (microbe-scale dispersal) and between locations through host movement (host-scale dispersal). In this paper, we examine how host- and microbe-scale spatial processes contribute to the spatial distribution of *Wolbachia* endosymbionts in *Aphaenogaster fulva-rudis-texana* (Hymenoptera: Formicidae) complex ants from Great Smoky Mountains National Park. We begin by identifying significant spatial variation in *Wolbachia* relative abundance at both the host (across the landscape) and microbe (across host lineages) scales. We then demonstrate a correlation between host- and microbe-scale environmental selection, complicating efforts to isolate the independent effects of host- versus microbe-scale processes. To overcome this challenge, we leverage both the broad distributions of individual host lineages across different environments and sites of co-occurrence between different host lineages within the same environments. This allows us to assess how both host- and microbe-scale processes contribute to spatial variation in our system. Ultimately, our results shed light on the myriad of interacting factors governing spatial variation in HA microbes and why spatial variation in HA microbes is more challenging to understand than spatial variation in free-living organisms.

## Introduction

Two of the most important drivers of a taxon’s spatial distribution are environmental selection and dispersal limitation.^1^ Environmental selection (i.e., taxon sorting) causes spatial structure by limiting access of certain taxa to certain locations based on their ability to tolerate local biotic or abiotic conditions. This can yield a wide array of different spatial patterns, including the evolution of ecotypes, genetic isolation by environment (IBE),^2,3^ and distance-decay of community similarity.^4^ Like environmental selection, dispersal limitation also causes spatial structure by limiting access of certain taxa to certain locations.^5^ In the case of dispersal limitation, however, access is limited by the ability of taxa to cross physical barriers and/or unsuitable habitat.^5^ Dispersal limitation can result in a wide variety of different spatial patterns, including genetic isolation by distance (IBD), metapopulation dynamics and, once again, distance-decay of community similarity.

While the spatial distributions of free-living organisms are primarily governed by environmental selection and dispersal limitation at the scale of the organism, the spatial distributions of host-associated (HA) microbes are governed by environmental selection and dispersal limitation at two scales: the scale of the microbe and the scale of their host. Thus, spatial variation in HA microbes is affected not only by dispersal of microbes from one host to another (microbe scale dispersal limitation) and differences in conditions on the host (microbe scale environmental selection; e.g., due to differences in host genetics or phenotype), but also by dispersal of hosts from one location to another (host scale dispersal limitation) and differences in conditions where the hosts reside (host scale environmental selection).^6,7^ This makes interpretation of HA microbial spatial patterns challenging. More specifically, because host-scale (i.e., variation in landscape-level environment and host movement) and microbe-scale (i.e., variation in host characteristics, transfer between hosts) processes are often correlated, it can be difficult to disentangle their independent effects on HA microbes. As an example, host genetic distance often correlates with host geographic distance and/or host environmental distance due to processes like genetic drift and local adaptation of the host.^8–10^ Consequently, the environment external to the host (host-scale) and the environment created by the host (microbe-scale) frequently co-vary, making it difficult to attribute spatial patterns to host- versus microbe-scale environmental selection.

Further complicating the interpretation of spatial patterns in HA microbes is the potential for bi-directional interactions across scales. Thus, just as host scale processes can impact the spatial ecology of HA microbes (i.e., top-down effects), microbe scale processes can influence the spatial ecology of their hosts (i.e., bottom-up effects).^11^ Many microbes, for instance, alter host dispersal, either as a side-effect of their impacts on host health^12^ or because they have evolved to manipulate host dispersal to promote their own transmission.^13,14^ HA microbes can also alter host environmental selection. In some systems, for example, it is the abiotic tolerances of obligate endosymbionts, rather than the hosts themselves that restrict environmental associations.^15^ Beyond dispersal and selection, HA microbes can also manipulate host reproduction. This is important to spatial patterns because it can impact host gene flow between regions.^16,17,18^ One of the most notorious examples is *Wolbachia*^18,19^ – a widespread^20^ bacterium that can affect host genetics by inducing cytoplasmic incompatibility (CI) among hosts harboring differing *Wolbachia* strains.^21,22,23,24^

Because of the complicated, correlated and often bi-directional nature of interactions between host- and microbe-scale processes, it can be difficult to determine the degrees to which processes at each scale influence the spatial structure of HA microbes. Regions where cross-scale correlations break down, however, can sometimes be useful to disentangle the effects of host- versus microbe-scale processes. In this paper, we investigate the roles of host- and microbe-scale spatial processes on spatial patterns in *Wolbachia* carriage among *Aphaenogaster fulva-rudis-texana* (Hymenoptera: Formicidae; henceforth ‘*A. rudis*’) complex ants in Great Smoky Mountains National Park (GSMNP). Leveraging previously derived, high resolution GSMNP bioclimatic variables,^25,26^ we assess the extent to which *Wolbachia* relative abundance varies as a function of the environment of the host (i.e., host scale environmental selection) and/or spatial distance (i.e., host scale dispersal limitation). We then assess the extent to which *Wolbachia* relative abundance varies as a function of host genetics (i.e., microbe scale environmental selection) and/or host mitotype (i.e., microbe scale dispersal limitation). Next, we show how host- and microbe-scale processes are strongly correlated, potentially complicating interpretation of spatial patterns. Finally, we repeat our analyses, subsetting our dataset in ways that allow us to break correlations between host- and microbe-scale processes. Ultimately, this allows us to identify the independent roles of processes at both scales as determinants of *Wolbachia* relative abundance in *Aphaenogaster* ants from GSMNP.

## Materials and Methods

### Study Species

Ants of the *A. rudis* complex are abundant and widespread in eastern hardwood forests of North America.^27^ With a generalist strategy that includes colonization of a wide array of forest types,^28^ and broad latitudinal,^29^ and elevational ranges,^30–34^ this complex is ideal for examining effects of host scale environmental selection on HA microbes. Further, *A. rudis* complex ants within GSMNP comprise four incipient species - *A. rudis*, *A. picea*, *A. fulva*, and *A. carolinensis*. Generally speaking, *A. picea* is found at higher elevations while *A. rudis* is found at lower elevations.^35^ Despite these differences,^35,36^ members of the complex co-occur extensively, both within GSMNP and across the Southeast broadly, ^37^ possibly because they are in the early stages of lineage divergence.^37^ This makes the *A. rudis* complex ideal for examining the effects of microbe scale environmental selection (i.e., host genetics) on HA microbes.

### Study Sites

All ants were collected from GSMNP. GSMNP is an ideal location for studying the effects of host scale environmental selection due to its mountainous terrain. In particular, the large elevational gradients characteristic of the GSMNP landscape result in significant bioclimatic variation over relatively small distances.^25,26^ *A. rudis* complex ants were collected from 12 All Taxa Biodiversity Index (ATBI) plots^38^ as well as an additional plot at one of the lowest elevations in the park (13 total sites) from Fall 2019 - Summer 2021. These plots vary greatly in elevation (522-1,494 meters) and were selected to represent the dominant ecosystems (i.e., distinct environments with different selection pressures) present in GSMNP. At each plot, we collected between 4 and 15 1m x 1m quadrats of litter across 6 to 11 unique time-points, depending on site accessibility. All quadrats within any sampling period were selected to be at least 5m apart to avoid sampling the same colony within multiple quadrats, and at most one ant from any quadrat was selected for sequencing. In addition to quadrat sampling, we spent ~ 30 minutes searching for and aspirating ants during each visit. (For additional details regarding ant collection and identification, see SI I).

#### COI and CAD Gene Extraction, Amplification, and Sequencing

Genomic DNA was extracted from 1 leg of each ant using the Thermo Scientific Genejet Genomic DNA purification kit following the manufacturer’s protocol. We amplified the COI gene following Harden et al. 2022^39^ using the primers LCO1490 and HCO2198 in 25-μL PCR reactions containing 2.5 μL of template DNA, 1.0 μL of each primer, 2.5 μL of dNTPs, 2.0 μL of MgCl2, 0.125 μL of Platinum Taq, 2.5 μL of buffer, and 13.375 μL of Invitrogen ultra-pure water (Catalog number: 10977023). Reactions were carried out using an Eppendorf MasterCycler with the following settings: initial denaturation stage of 180 seconds at 95°C followed by 35 cycles of a denaturation stage at 94°C for 30 seconds, an annealing stage at 45°C for 30 seconds, an extension stage at 72 °C for 45 seconds, and ending with a final extension at 72°C for 180 seconds. CAD was amplified using primers CD439F and CD851R.^51^ CAD reactions were carried out as above using an Eppendorf MasterCycler beginning with an initial denaturation at 95°C for 3 minutes followed by 40 cycles of a denaturation stage at 94°C for 30 seconds, an annealing stage at 50°C for 30 seconds, an extension stage at 72°C for 50 seconds, and a final extension at 72°C for 5 minutes. Amplified genes were forward and reverse Sanger sequenced at Psomagen.

#### COI and CAD Phylogenetic Tree Construction

Forward and reverse COI and CAD Sanger sequencing reads were merged in R (v.4.2.1)^40^ using the package sangeranalyseR (v.1.6.1).^41^ After quality control, 91 CAD sequences and 80 COI sequences remained from our original 102 samples. We aligned merged reads, without a reference, using our own data as well as data from an *A. umphreyi* individual that we chose as our outgroup (GenBank Accessions: KP860492.1 and KP730141.1).^42^ COI and CAD genes were aligned separately using the MAFFT algorithm in Mesquite. We then employed maximum likelihood methods for phylogenetic tree construction through the web server version (v. 2.4.0) of IQ-Tree^43^ using default parameters. This was repeated for the COI and CAD single alignments separately to produce single gene trees. For the COI (mtDNA marker) and CAD (nuDNA marker) genes, the best-fit models of nucleotide substitution, selected based on BIC and using ModelFinder within IQ-Tree, were TPM2u+F+R2 and K2P+R2 respectively. Tree discordance between COI and CAD gene trees was calculated using the *cospeciation* function and visualized using the *cophylo* function both in the *phytools* package in R (version 4.2.1). All statistics were performed using separate COI and CAD trees rather than a concatenated tree. For host-specific analyses, we used all COI and/or CAD sequences available (80 and 91 samples respectively). For analyses that paired COI/CAD and *Wolbachia*, we only used samples for which both host and *Wolbachia* data were available (46 samples for COI/*Wolbachia*, 52 samples CAD/*Wolbachia*, 41 samples COI/CAD/*Wolbachia*). For all analyses, we used a square-root transformation of branch lengths^44^ in either the COI or CAD trees.

#### Extraction, Amplification, and Sequencing of Microbial DNA

Microbial DNA was extracted using the ZymoBIOMICS DNA Microprep kit (catalogue number D3401). Prior to extraction, individual ants were surface sterilized for 1 minute in 10% bleach solution and rinsed 3 times in ultra-pure water to ensure transient microbes on the exoskeleton were removed. We performed the extraction following the manufacturer’s protocol with the following modifications: 600 μL input of ant lysate was added to 1800 μL of binding buffer due to low input. We skipped filtering steps 4, 12, and 13 due to low input and heated the elution buffer to 60°C prior to elution to maximize DNA concentrations. Samples were lysed for 40 minutes using a vortex genie and for 20 minutes using a tissuelyzer2 (Qiagen) at 25 x/second to effectively lyse the ant exoskeleton.

We characterized entire bacterial communities by amplifying the V4 region of the 16S rRNA gene using touchdown PCR with common dual index primers.^45^ Briefly, we amplified in 20-μL PCR reactions containing 5 μL DNA, 5 μL of the primer set (515F and 806R), 2 μL of 10x Accuprime PCR Buffer II, 0.15 μL of Accuprime HiFi polymerase, and 7.85 μL of ultra-pure water per reaction. Reactions were carried out using an Eppendorf MasterCycler with the following settings: an initial denaturation stage of 120 seconds at 95°C followed by 20 cycles of a denaturation stage at 95°C for 20 seconds, an annealing stage at 60 °C for 20 seconds, and an extension stage at 72°C for 5 minutes. This was followed by 20 cycles of a denaturation stage at 95°C for 20 seconds, an annealing stage at 55°C for 20 seconds, an extension stage at 72°C for 5 minutes, and a final extension stage at 72°C for 10 minutes. Samples were normalized prior to pooling using a SequalPrep Normalization Plate Kit (catalogue number #A1051001). Pooled sample library concentrations were quantified via KAPA qPCR (Roche, cat. #07960140001). Multiplexed pooled libraries were sequenced paired end 2 x 300 cycles on an Illumina NextSeq 2000 to an average depth of 245,448 reads. Resulting FASTQ files were analyzed using a custom Qiime2^46^ pipeline. (For additional information on Taxonomic Assignment and Dataset Manipulation, see SI I; see also Github repository below).

#### Environmental axis

We considered elevation, mean soil moisture (volumetric content), soil minimum temperature, and soil maximum temperature derived from previous GSMNP studies.^25,26^ We first z-transformed these four environmental variables, and then calculated environmental distances based on the Euclidean distances between the z-transformed environmental variable values at each capture location. Next, we performed a principal component analysis (PCA) based on the environmental variables associated with each ant. We then selected the first principal component (PC1; i.e., the axis capturing the most variation in the system) and used the value of each ant along this axis for subsequent analysis of environmental effects.

#### Statistical Analyses

All statistical analyses were performed in R (v.4.2.1).All data and code can be found in the following github repository: https://github.com/dmalago/Aphaenogaster_GSMNP_Microbiome.

##### Spatial autocorrelation:

We used the *jitter* function from base R tojitter site values for latitude and longitude so that each ant sample had a unique location. We then used the *chooseCN* function from the *adespatial* package to create a connection network. This was done using Delaunay triangulation (type 1). From this network, we used the *nbdists* function from the *spdep* package to calculate edge distances. Next, we used the *nb2listw* function from the *spdep* package to find row-standardized (style = 4) spatial weights for network edges. Finally, we inputted *Wolbachia* relative abundances and the list of spatial weights into the *moran.test* function, also from the *spdep* package, to determine whether there was significant autocorrelation. Results were visualized using the *moran.plot* function, again from the *spdep* package.

##### Regression Analyses:

We used generalized linear models^47^ with beta distributions to regress *Wolbachia* relative abundances against principal component one (PC1) of the environmental PCA (*Wolbachia ~ Environment*, see above for axis definition). This was done for all ants and for ants from individual lineages separately. We also regressed *Wolbachia* relative abundance against host COI and CAD clade/group (*Wolbachia* ~ COI_Clade_*CAD_Clade_, see Results for information on clades/groups). All regressions were performed using the *betareg* function from the *betareg* package.

##### Heatmaps:

We constructed heatmaps of *Wolbachia* relative abundance for each *Wolbachia* ASV in each ant using the *pheatmap* function from the *pheatmap* package. This was done ordering ants according to both the COI and the CAD phylogenies.

##### Mantel tests:

We used Mantel^48^ tests to assess whether host relatedness varied with geographic distance and whether host relatedness varied with environmental similarity. All Mantel tests were performed using the *mantel* function from the *vegan* package(v. 2.6.4).^49^ We then used the *mantel.correlog* function, also from the *vegan* package (v. 2.6.4), to calculate mantel correlations at varying geographic or environmental distance classes. For spatial distance, we used the Haversine formula to calculate the great circle distance between all ant capture locations based on jittered (see above) longitude and latitude. This was implemented using the *distm* function from the *geosphere* package (v. 1.5-20). For environmental distance, we considered the distance between ants along our PC1 environmental axis (see Environmental axis, above). For host relatedness, we used distances based on the branch lengths of the square root transformed COI or CAD trees. Branch lengths were calculated using the *cophenetic.phylo* function from the *ape* package (v. 5.7.1).^50^

##### Paired sample Wilcoxon tests:

We used paired sample Wilcoxon tests^51^ to test for differences in *Wolbachia* relative abundance between host clades/groups (see Results), controlling for environment/spatial autocorrelation. First, we identified sites where different COI clades/groups were co-localized. We then averaged *Wolbachia* relative abundances for all ants from each clade/group at each site of co-localization. This gave a single *Wolbachia* relative abundance for each clade/group at each site. We then used these average *Wolbachia* relative abundances in paired sample Wilcoxon tests, pairing ants from different clades/groups at the same sites.

## Results

### Ant Clades/Groups

Using the COI consensus tree (Fig. S2.1 – left side), we visually identified 2 main clades (henceforth the “blue” and “purple” clades) as well as a set of basal ants (henceforth the “red” [paraphyletic] group, see also SI Figure S3.6B for haplotype maps suggesting these are a separate group but not necessarily basal to the blue and purple clades). Using the CAD consensus tree (Fig. S2.1 – right side), we visually identified two main clades (henceforth the “green” and “yellow” clades) as well as several basal ants (henceforth the “black” group). Although consensus trees for COI and CAD sequences displayed significantly different topologies (RF distance = 148, p = 1), there was a relatively high degree of congruence between the red, purple and blue COI clades/groups and the green and yellow CAD clades. In particular, ants with red group COI sequences were significantly more likely to have green clade CAD sequences, while ants with blue clade COI sequences were significantly more likely to have yellow clade CAD sequences (Fisher’s exact test: *p* = 0.00067, see SI II Figure S2.2, Table S2.1).

### Ant Microbiota

Based on regression analyses (see Statistical Analyses), *Wolbachia* was the only microbial taxon whose relative abundance varied significantly across either environment or host clade (see SI III Figures S3.1 and S3.2). Consequently, we chose to focus on elucidating spatial patterns in *Wolbachia* carriage. Thirty-two separate ASVs were taxonomically classified to the *Wolbachia* genus, including members of supergroups A, B and F (see SI III Figure S3.3).^18^ Only three, however, independently comprised more than 0.01% of reads across all ant microbiota (two from supergroup A, and one from supergroup F). These were also the only *Wolbachia* ASVs found in more than one ant.

### Host-scale variation in Wolbachia

**Figure 1:**
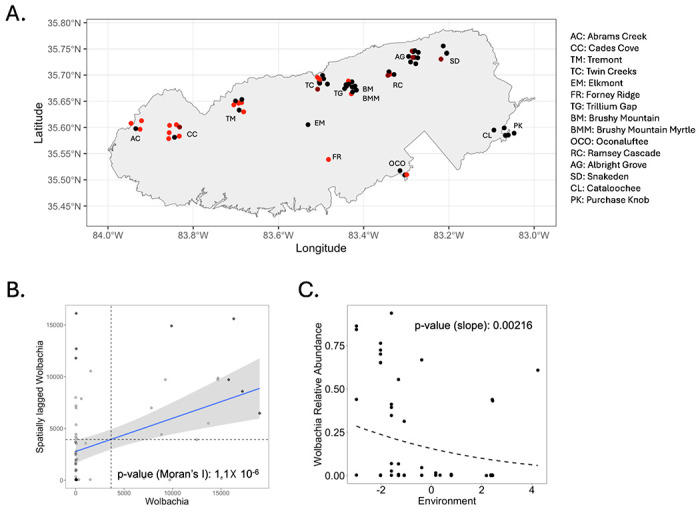
(A) *Wolbachia* relative abundance (black: <0.5%, dark red: >0.5% and < 5%, red: >5%) for all 68 *Aphaenogaster* microbiomes shown based on site of collection (note that site coordinates are jittered to make all samples visible); (B) Moran’s I scatterplot for *Wolbachia* relative abundance; (C) Beta regression model of *Wolbachia* relative abundance as a function of the PC1 environmental axis.

*Wolbachia* relative abundances exhibited significant spatial variation across GSMNP (see Figure 1). Whereas *Wolbachia* was present on most ants from the western side of the park, *Wolbachia* was absent or present in very low abundances (<0.05%, see Figure 1A) on most ants from the eastern side of the park. This resulted in significant positive spatial autocorrelation in *Wolbachia* relative abundance (see Figure 1B). Regressing *Wolbachia* relative abundance against the PC1 environmental axis further indicated that *Wolbachia* relative abundance was higher in ants from warmer, drier, lower elevation regions of the park and that *Wolbachia* relative abundance was lower in ants from cooler, wetter, higher elevation regions of the park (see Figure 1C). This was consistent with overall spatial trends, since the western side of the park is generally warmer, drier and lower elevation than the eastern side of the park. Using variance partitioning to separate the spatial versus environmental drivers of *Wolbachia* abundance, we found that both spatial distance (9% of variation) and the interaction between spatial distance and environment (16% of variation) were important for explaining differences in *Wolbachia* relative abundances across ants (see SI III Figure S3.5).

### Microbe-scale variation in Wolbachia

**Figure 2:**
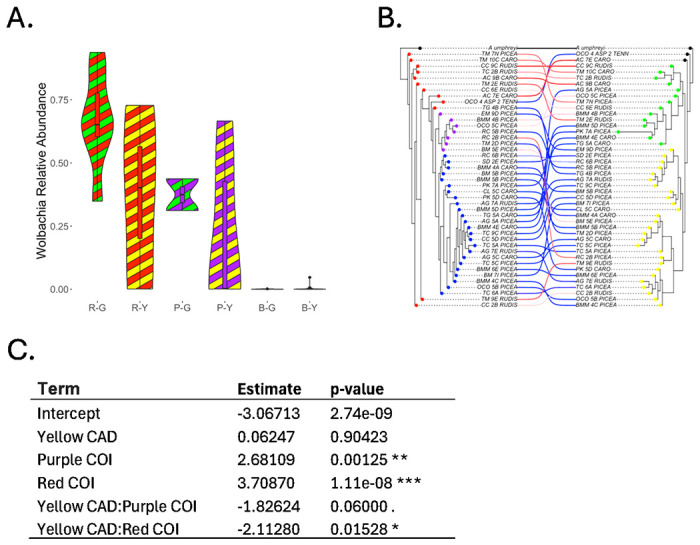
(A) Violin plot of *Wolbachia* relative abundance for ants with the red clade COI sequence and the green clade CAD sequence (R-G, red/green), the red clade COI sequence and the yellow clade CAD sequence (R-Y, red/yellow), the purple clade COI sequence and the green clade CAD sequence (P-G, purple/green), the purple clade COI sequence and the yellow clade CAD sequence (P-Y, purple/yellow), the blue clade COI sequence and the green clade CAD sequence (B-G, blue/green) and the blue clade COI sequence and the yellow clade CAD sequence (B-Y, blue/yellow); (B) cophylogeny of the *Aphaenogaster* COI sequences (left) and CAD sequences (right); COI (red, purple, blue) and CAD (green, yellow, black) clades are shown based on the color of the leaf nodes, while *Wolbachia* relative abundance (blue: low, red: high) is shown based on the color of the links; (C) results of a beta regression for the model Wolbachia ~ COI_clade*CAD_clade.

**Figure 3:**
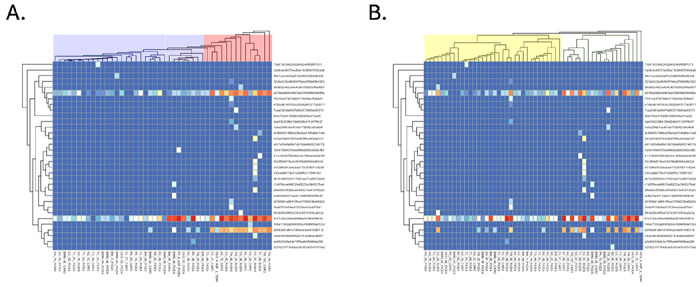
Heatmaps showing the number of reads of each *Wolbachia* ASV on each ant, organized according to host COI (A) and CAD (B) phylogenies and the *Wolbachia* phylogeny. The color scheme is logarithmic. COI and CAD clades are colored on their respective heat maps.

*Wolbachia* relative abundance varied significantly with both mitochondrial and nuclear sequence variation of the host (see Figure 2). More specifically, our regression model suggested that ants with red group and purple clade COI sequences had significantly higher *Wolbachia* relative abundances as compared to ants with blue clade COI sequences, and that ants with red group and (marginally) purple clade COI sequences had significantly lower *Wolbachia* relative abundances when they had yellow as compared to green clade CAD sequences. Closer inspection suggested that three distinct *Wolbachia* ASVs were responsible for the majority of *Wolbachia* in our system (see Figure 3, see also SI III figure S3.6). Two of the three dominant ASVs (henceforth wArudA1, wArudA2) were closely related, clustering together insupergroup A and differing from each other by 1/214 base pairs (bp). By contrast, the third dominant ASV (henceforth wArudF1) was more distantly related, clustering in supergroup F and differing from wArudA1 and wArudA2 by 8/214 and 9/214 bp respectively (see SI III, Figure S3.3). Notably, these three ASVs showed strong variation across COI ant clades, indicating a likely link between mitotype and the presence of specific *Wolbachia* variants (see Figure 3A, see also SI III Figures S3.6B and S3.7). In particular, whereas ants from the red clade had all three *Wolbachia* ASVs, ants from the purple clade had only wArudA2, and ants from the blue clade had none of the three *Wolbachia* ASVs. As compared to host COI sequences, the presence/absence of individual *Wolbachia* ASVs was not as strongly correlated with host CAD sequences. Nevertheless, consistent with the lower abundance of *Wolbachia* in yellow clade ants overall, a larger proportion of green clade ants had all three *Wolbachia* ASVs, while a larger proportion of yellow clade ants had no *Wolbachia* ASVs (see Figure 3B, see also SI III Figures S3.6C and S3.8).

### Correlation between host- and microbe-scale drivers of variation

**Figure 4:**
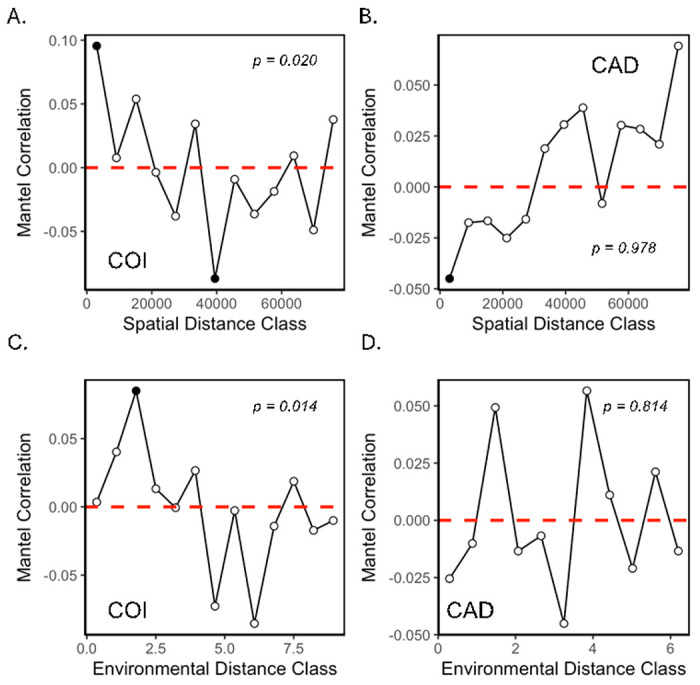
Mantel correlograms for sequence similarity based on the COI (A, C) and CAD (B, D) consensus trees, regressed against spatial distance (A, B) and environmental distance (C, D) and assessed with Spearman’s rank correlation. *p*-values shown on each panel reflect the significance of the corresponding Mantel tests (see also SI II Figures S2.2 and S2.3).

**Figure 5:**
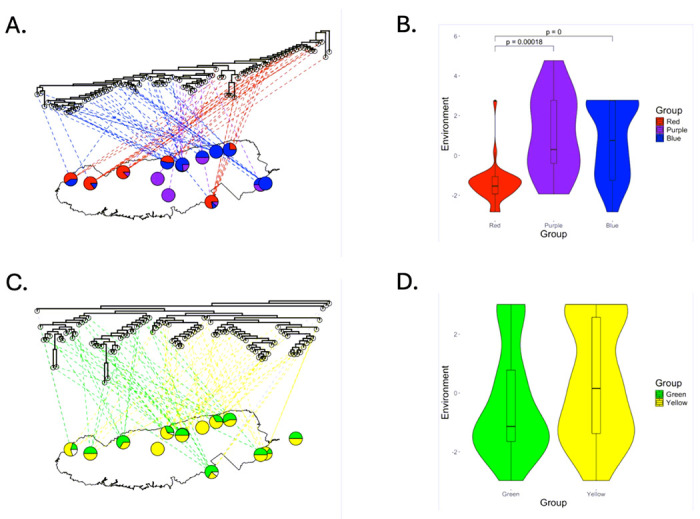
COI (A) and CAD (C) phylogenetic trees mapped to 14 sites of origin across 80 ants (A) and 92 ants (B) in GSMNP using the *plot.phylo.to.map* function in the phytools package. The node unassociated with a GSMNP location is the outgroup, *Aphaenogaster umphreyi*. Lines are colored based on whether the ant is included in the (A) red, blue or purple clades and (C) green, yellow or basal (white) clades. Pie charts show the fraction of ants at each location that come from each COI or CAD clade. (B, D) Violin plot showing comparison of values along the conglomerate environmental axis for red clade, blue clade and purple clade ants (B) and for the green and yellow clade ants (D).

Mantel tests based on Spearman, but not Pearson correlation coefficients demonstrated a significant relationship between COI sequence similarity and geographic distance (Spearman: r: 0.1005, p = 0.020; Pearson: r = 0.0703; p = 0.082; Figure 4A, see also SI II Figure S2.3A,B). Likewise, Mantel tests based on both Spearman and Pearson correlation coefficients demonstrated a significant relationship between COI sequence similarity and environmental similarity (Spearman: r = 0.0912, p = 0.014; Pearson: r = 0.0685; p = 0.042; Figure 4C, see also SI II Figure S2.3C,D). Distance-based redundancy analysis (dbRDA) and variance partitioning supported results from individual Mantel tests, suggesting that both distance and distance + environment explained the similarity of COI sequences (see SI II Figure S2.5A). Consistent with the observed correlations between COI sequence similarity and both spatial and environmental distance, ants with red group, purple clade and blue clade COI sequences exhibited different distributions across GSMNP. Red group ants dominated lower elevations on the western side of the park, while blue clade ants dominated higher elevations on the eastern side of the park. Purple clade ants were predominantly found towards the center of the park where they co-occurred with the other two clades/groups across a range of elevations (see Figure 5A). In accordance with their differing distributions, environmental conditions were also significantly different across the three COI clades/groups (see Figure 5B; Kruskal-Wallis chi-squared = 26.681, df = 2, p-value = 1.6E-6, see also SI II Figure S2.6A). Ants from the red group were associated with warmer, drier, and lower elevation conditions while ants from the blue clade were associated with colder, moister and higher elevation conditions. Ants from the purple clade were intermediate, though differences between the blue and the purple clades were not significant.

Unlike COI sequences, no significant relationships were observed between CAD sequence similarity and either geographic distance (Spearman: r: −0.0902, p = 0.978; Pearson: r = −0.0634 ; p = 0.882; Figure 4B, see also SI II Figure S2.4A,B) or environmental distance (Spearman: r = −0.0319, p =0.814 ; Pearson: r = −0.0379, p = 0.832; Figure 4D, see also SI II Figure S2.4C,D). Likewise, CAD sequences did not exhibit obviously different distributions across GSMNP (see Figure 5C), nor were environmental conditions significantly different between the green and yellow clades (see Figure 5D, Kruskal-Wallis chi-squared = 2.0236, df =1, p-value = 0.1549; see also SI II Figure S2.6B).

### Isolating host-scale drivers of variation in Wolbachia

**Figure 6:**
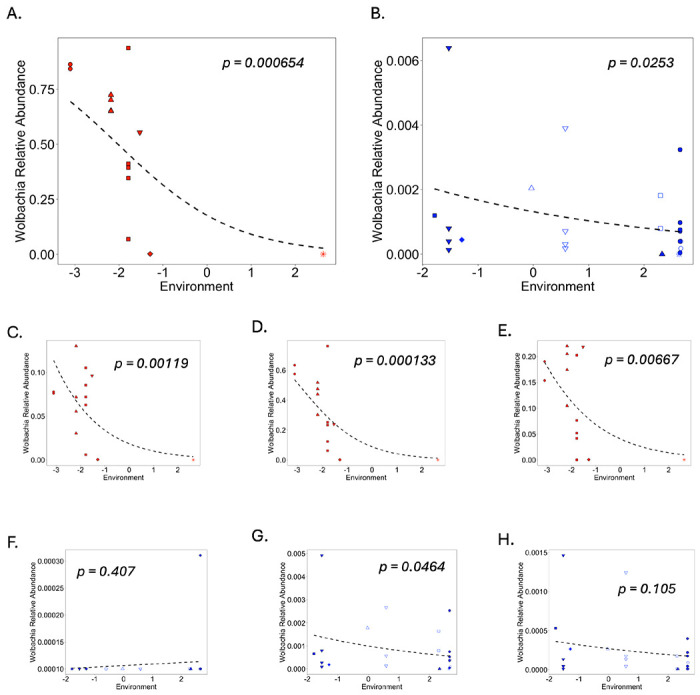
Beta regression models of total (A,B) *Wolbachia* relative abundance and relative abundances of the wArudA1 (C,F), wArudA2 (D,G) and wArudF1 (E,H) ASVs as a function of our conglomerate environmental variable (see Methods) for (A,C-E) red clade ants and (B,F-H) blue clade ants. p-values indicate significance of the environment, and specific sites are denoted as follows: Abram’s Creek (closed red circles), Tremont (closed upward red triangles), Cades Cove (closed squares), Twin Creeks (closed downward triangles), Oconaluftee (closed diamond), Trillium Gap (star), Snake Den (open upward triangle), Albright Grove (open downward triangle), Cataloochee (closed upward blue triangles), Purchase Knob (open squares), Brushy Mountain Myrtle (closed blue circles), Brushy Mountain (open circles).

We examined the role of environment (host-scale process) while controlling for COI sequence (microbe-scale process) by considering the effects of the PC1 environmental variable on *Wolbachia* carriage by blue clade and red group ants separately. When we did this, we found that overall *Wolbachia* abundance, as well as abundance of each of the three dominant *Wolbachia* ASVs (wArudA1, wArudA2, and wArudF1) varied in response to environment (see Figure 6A,C–E) for red group ants. In particular, there was a precipitous decline in *Wolbachia* at a threshold environmental PC1 value of approximately −2 (roughly corresponding to a minimum soil temperature of −5°C, a soil moisture of 0.073 % volumetric moisture content, a soil temperature range of 32.5°C and an elevation of 622 m) with the two ants from the two coldest sites (Oconaluftee (OCO), Trillium Gap (TG)) exhibiting much lower (<0.5%) *Wolbachia* relative abundance as compared to ants from other sites. While the ant from Trillium Gap was yellow clade for its CAD sequence (i.e., potentially lower *Wolbachia* due to host genetics), the ant from Oconaluftee had a basal (‘black group’) CAD sequence. Unlike red group ants, the relationship between *Wolbachia* and environment was less apparent for blue clade ants, only reaching significance for overall *Wolbachia* relative abundance and the abundance of the wArudA2 ASV and, even then, only reaching significance when an outlier ant from Ramsey Cascades was removed. Notably, this was a generally unusual ant containing both wArudA2 and wArudF1 (most other ants either contained all three *Wolbachia* ASVs or else only wArudA2, see SI III Figure S3.6). The weaker relationship between *Wolbachia* and environment in blue clade ants is not surprising given the dramatically lower *Wolbachia* relative abundances in blue clade ants in general. Overall, within-clade comparisons, particularly for red group ants, suggest that host-scale processes (environmental conditions and/or host dispersal limitation) independently impact the spatial distribution of *Aphaenogaster*-associated *Wolbachia* across GSMNP.

### Isolating microbe-scale drivers of variation in Wolbachia

We examined the effects of COI sequence (microbe-scale process) while controlling for environment (host-scale process) by contrasting average *Wolbachia* carriage of ants from different COI clades/groups paired according to collection site. When we did this, we found that red group and blue clade ants co-occurred at four sites, and that the difference in *Wolbachia* carriage was borderline in significance. Likewise, we found that red group and purple clade ants and purple clade and blue clade ants co-occurred at two and three sites respectively, and that the differences in *Wolbachia* carriage were not significant. Finally, we found that red group + purple clade and blue clade ants co-occurred at seven sites, and that the difference in *Wolbachia* carriage was highly significant (see Table 1). In addition, we examined the effects of CAD sequence (microbe-scale process) while controlling for environment (host-scale process) by contrasting *Wolbachia* carriage across ants from different CAD clades paired according to collection site. Again, when we did this, we found that green and yellow clade ants co-occurred at nine sites, and that the difference in *Wolbachia* carriage was significant. Finally, we examined the effects of COI/CAD sequence while controlling for environment. In this case, we found that red + purple/yellow clade ants and red + purple/green clade ants co-occurred at four sites, and the difference in *Wolbachia* carriage was not significant. Overall, these comparisons across paired samples suggest that microbe-scale processes (host genetics and/or host-to-host transmission), particularly processes correlated with COI sequence, independently impact the spatial distribution of *Aphaenogaster*-associated *Wolbachia* across GSMNP.

## Discussion

In this paper, we examined host- and microbe-scale patterns in *Wolbachia* relative abundance among *A. rudis* complex ants from across Great Smoky Mountains National Park (GSMNP). Our goal was to determine whether spatial structure was primarily attributable to host-scale processes, microbe-scale processes or a combination of the two (i.e., multiscale processes).

### Independent effects of host-scale processes

We inferred independent effects of host-scale processes – more specifically, host-scale environmental selection – based on how environment impacted *Wolbachia* load across individual ant COI clades/groups. In particular, we found that some combination of low temperature, high moisture and high elevation lowers *Wolbachia* abundance/persistence in our system (see below). This is not unprecedented.^52,53^ In a recent study of captive *A. rudis* colonies, for example, *Wolbachia* relative abundance decreased in response to artifcial warming.^54^ While this trend is opposite what we observed, it nonetheless indicates that temperature can affect *Wolbachia* abundance. Indeed, differences in directionality could merely refect the existence of an optimum in the *Wolbachia* thermal performance curve. Notably, ants in the captive study were warmed to a constant temperature of 32°C, which is much higher than the ambient annual temperatures experienced by even the lowest elevation ants in our system (though not necessarily higher than the maximum annual temperature at the warmest sites). Thus, our range of temperatures may have been below the optimal growth temperature for *Aphaenogaster*-associated *Wolbachia*, causing an increase in *Wolbachia* with temperature. By contrast, the captive study may have heated ants past the optimal growth temperature, causing a decrease in *Wolbachia* with temperature. This is in keeping with studies of other Hymenoptera species^55^ which have demonstrated reduced *Wolbachia* carriage (e.g., due to temperature dependent interactions with a *Wolbachia* phage) at both excessively low and excessively high temperatures. Interestingly, a recent meta-analysis^56^ across ~2500 species of arthropods also hints at the possibility of a unimodal response of *Wolbachia* to temperature, with *Wolbachia* prevalence increasing with temperature in temperate zones (i.e., from a lower baseline temperature) and decreasing with temperature in tropical zones (i.e., from a higher baseline temperature).

While the host-scale patterns that we observed are well explained by host-scale environmental selection, they are not as easily explained by host-scale dispersal limitation. If host-scale dispersal limitation was fully responsible for the spatial distribution of *Aphaenogaster*-associated *Wolbachia* in GSMNP, then, in keeping with our findings, there should be fewer *Wolbachia*-positive ants on the eastern side of the park. However, *Wolbachia*-positiveants that *do* reach the eastern side of GSMNP should have similar *Wolbachia* loads to their *Wolbachia*-positive counterparts on the western side of the park(i.e., the frequency of *Wolbachia*-positive ants should decrease, but the relative abundance of *Wolbachia* on *Wolbachia*-positiveants should be similar everywhere). The fact that both prevalence and load decreased along the West-to-East gradient indicates that host dispersal is not the sole driver of *Wolbachia* spatial patterns. That said, host-scale dispersal limitation does seem to exist, at least based on COI sequence (see SI II Fig. S2.5A) and thus, could partially explain the smaller fraction of *Wolbachia*-positive ants on the East side of the park - even if it doesn’t explain the overall lower *Wolbachia* loads in eastern ants when *Wolbachia* is present.

### Independent effects of microbe-scale processes

We inferred independent effects of microbe-scale processes based on the significantly different *Wolbachia* loads across host mitotypes, even in paired samples from the same sets of sites. Notably, this pattern could have emerged either through microbe-scale environmental selection or microbe-scale dispersal limitation. Microbe-scale environmental selection would indicate that the mitochondrial COI sequence is correlated (not necessarily causally) with some aspect of host biology (e.g., immune system, behavior, food preference) that impacts *Wolbachia* performance. Again, this would not be unprecedented. Among leaf-cutter ants (*Acromyrmex echinatior*), for example, different patrilines exhibit significant differences in *Wolbachia* densities.^57^ Meanwhile among *Vollenhovia emeryi*, genotypes associated with different wing morphology exhibit different *Wolbachia* colonization rates.^58^ Even among *Aphaenogaster*, a recent study identified differences in *Wolbachia* abundances between *A. rudis* and *A. fulva* ants, although these results were based on only three *A. fulva* and two *A. rudis* colonies.^59^

The alternate explanation – that *Wolbachia* are limited to red group ants because of microbe-scale dispersal limitation – would indicate a barrier to *Wolbachia* transmission among different host mitotypes. Again, this is consistent with what we know about *Wolbachia* biology.Broadly speaking, *Wolbachia* has two modes of microbe-scale dispersal: horizontal transmission and vertical transmission. Although extensive evidence exists suggesting that *Wolbachia* can be acquired horizontally across large host phylogenetic distances,^60^ including among ants,^61^ horizontal transmission within species requires a number of relatively restrictive conditions.^62^ This likely limits the extent to which *Wolbachia* can transfer directly from one ant to another (microbe-scale dispersal limitation), particularly over shorter, ecological timescales. Consequently, in our system, microbe-scale dispersal of *Wolbachia* likely occurs predominantly via vertical transmission (mother to offspring).^18^ As a result, *Wolbachia* colonization should closely mirror patterns in mtDNA inheritance, even if there is no correlation between mitochondrial COI sequence and other aspects of host biology impacting *Wolbachia* fitness (i.e., no microbe-scale environmental selection). Unfortunately, in our system, it is difficult to determine whether microbe-scale environmental selection, microbe-scale dispersal limitation or a combination of both underlies differences *Wolbachia* carriage across COI sequences. Nevertheless, the extremely tight coupling between COI sequence and each of the three *Wolbachia* ASVs, along with the limited number of *Wolbachia* acquisitions and losses predicted by ancestral state reconstructions (ASRs, see SI III, Figures S.3.7 and S3.8), suggests that dispersal is strongly limited to vertical transmission. This makes microbe-scale dispersal limitation a strong contender for explaining the restriction of *Wolbachia* to red group ants and, to a lesser extent, purple clade ants. Consistent with this hypothesis, we did not find any relationships between other bacterial taxa (including both other endosymbionts and ectosymbionts), and COI sequence (see SI III, Figures S3.1 for visualizations), although it is possible that some of the other putative endosymbionts (e.g., *Sulcia*)were only detected because they were present in prey from *Aphaenogaster* stomach contents.

Like COI sequence, CAD sequence also appears to be correlated with some aspect of host biology that impacts *Wolbachia* performance. Notably, we found an effect of CAD sequence even within COI clades, suggesting that the correlation between CAD sequence and *Wolbachia* is not fully explained by the same drivers that underlie the correlation between COI sequence and *Wolbachia*. Likewise, CAD sequence remained correlated with *Wolbachia* load even when controlling for environment, as would be expected given that CAD is not, itself, correlated with environment. Unfortunately, the relatively few western locations harboring red clade ants, coupled with the low capture rate of red clade ants at eastern locations, made it difficult for us to test the effect of CAD within COI clades while simultaneously controlling for environment. Rather, we had only four sites for our paired Wilcoxon test, which did not, as a result, rise to the level of significance. Nevertheless, there was still evidence of a weak trend, suggesting that, with additional statistical power, it might be possible to detect independent effects of COI and CAD independent of environment (see Table 1). Regardless of whether COI and CAD sequences reflect the same or different aspects of host biology underlying *Wolbachia* load, there is no question that microbe-scale processes are an independent contributor to the spatial distribution of *Aphaenogaster*-associated *Wolbachia* across GSMNP.

### Possible interactions between host and microbe scales

While we found conclusive evidence for independent effects of both host- and microbe-scale processes driving patterns in *Aphaenogaster*-associated *Wolbachia* abundance across GSMNP, we also identified ample opportunity for multiscale interactions. Admittedly, such interactions are more difficult to demonstrate. Nevertheless, it is worth speculating on potential mechanisms that might connect host- and microbe-scale processes. Based on recent findings in *Drosophila*, for example, it is possible that colder host environments (i.e., host-scale environmental selection) reduce vertical transmission (i.e., microbe-scale dispersal limitation) of *Wolbachia* to *Aphaenogaster* alates.^63^ This could explain the lower prevalence (though not necessarily the lower abundance) of *Wolbachia* in ants, including red group ants, from the colder Eastern regions of the park. Another possible multiscale interaction is reproductive manipulation. If *Wolbachia* has been prevalent on the western side of the park for a long time, and if *Wolbachia* induces CI or some other form of reproductive manipulation in *A. rudis* ants, then this could prevent western invasion of blue clade COI, even as blue clade nuclear genes (i.e., yellow clade CAD) become incorporated into red group populations (i.e., the microbe alters host-scale gene flow). Consistent with this hypothesis, we observed a higher percentage of red group COI (30%) mismatched with yellow clade CAD as compared to blue clade COI (21%) mismatched with green clade CAD. This is what would be expected if the offspring of uninfected females and infected males are non-viable but the offspring of infected females and uninfected males are viable. Further, while mismatch in blue clade COI is unexpected, this may be explained by mismatch that existed prior to *Wolbachia* colonization (akin to incomplete lineage sorting) or as a result of incomplete CI. Notably, temperature differences can result in differing levels of both CI penetrance and *Wolbachia* density,^55,64,65^ providing a mechanism by which CI might serve as a stronger barrier to gene flow on the western side of the park, but a weaker barrier on the eastern side of the park. Another potential multiscale mechanism that may contribute to our findings is an effect of *Wolbachia* on ant dispersal. This could occur either because *Wolbachia* alters the movement patterns of alates or because it reduces production of alates entirely.^66^ In both cases, the effect would be to slow the eastern spread of red group COI, even if CI gives the red group a long-term competitive advantage.^67^ Finally, *Wolbachia* could prevent red group ants from penetrating eastward by lowering survival in the colder, wetter, higher elevation regions of the park. Again, this multiscale interaction could restrict the spatial extent of the red group COI, even if it gains a competitive demographic advantage through CI.

## Conclusions

The complex genetic structure of *A. rudis* ants, their propensity to inhabit a wide range of different environments across large spatial extents, and their colonization by multiple strains of the notoriously complex *Wolbachia* endosymbiont makes *A. rudis* an ideal system for studying the interplay between environmental selection and dispersal limitation at the host and microbe scales. Our findings suggest that this interplay leads to interesting spatial patterns across the mountainous terrain in GSMNP. Further, multiple opportunities exist for extending the current study. This includes better characterizing *Wolbachia* differences in the contact zone between COI clades/groups, undertaking more extensive comparisons of *Wolbachia* carriage in ants with and without COI/CAD mismatch and extending similar analyses from GSMNP to the Southern Appalachians more broadly in order to better tease apart the effects of environment versus distance in shaping patterns of *Wolbachia* prevalence and abundance. Beyond field characterization, another fruitful avenue would be lab studies examining how *Wolbachia* impacts host thermal tolerance, how temperature effects *Wolbachia* load and transmission, or even whether and how *Wolbachia* induces CI or other forms of reproductive manipulation.

As keystone mutualists, *Aphaenogaster* ants have garnered significant attention across various research areas,^27,29,33,42,68–72^ including recent explorations into their microbial associations.^59,73,74^ Our study suggests that, even beyond their importance to processes like seed dispersal and pest control,^75^ *A. rudis* complex antsmight serve as model systems for understanding the multiscale, bidirectional interactions between hosts and their HA microbes.

## Supplementary Material

This is a list of supplementary files associated with this preprint. Click to download.

Malagonetal092525SI.docx

## Figures and Tables

**Table 1.** Results of paired sample Wilcox tests comparing average relative abundance of *Wolbachia* from ants where red, purple and/or blue clade ants co-occur.

Comparison	V-statistic	p-value
blue:red	10	0.0625 .
blue:purple	9	0.125
purple:red	2	0.5
red+purple:blue	28	0.007813**
yellow:green	37	0.049*
red+purple/yellow:red+purple/green	9	0.125

## Data Availability

The datasets generated and/or analysed during the current study are available in the National Center for Biotechnology Information (NCBI) sequence read archive (SRA) under project number PRJNA1347072. All data and code (BIOM files, metadata files, and all R code) necessary for the analyses presented in this manuscript are additionally available at https://github.com/dmalago/Apheanogaster_GSMNP_Microbiome. Research results have been shared with Great Smoky Mountains National Park through yearly reports.
